# Vertically Aligned Binder-Free TiO_2_ Nanotube Arrays Doped with Fe, S and Fe-S for Li-ion Batteries

**DOI:** 10.3390/nano11112924

**Published:** 2021-10-31

**Authors:** Suriyakumar Dasarathan, Mukarram Ali, Tai-Jong Jung, Junghwan Sung, Yoon-Cheol Ha, Jun-Woo Park, Doohun Kim

**Affiliations:** 1 Nano Hybrid Technology Research Center, Electrical Materials Research Division, Korea Electrotechnology Research Institute, Changwon 51543, Korea; suriyakumar@keri.re.kr (S.D.); swg9311@keri.re.kr (J.S.); 2 Department of Electro-Functionality Materials Engineering, University of Science and Technology (UST), Daejeon 305-333, Korea; mali@keri.re.kr (M.A.); tjjung@keri.re.kr (T.-J.J.); 3 Next Generation Battery Research Center, Electrical Materials Research Division, Korea Electrotechnology Research Institute, Changwon 51543, Korea; ycha@keri.re.kr (Y.-C.H.); parkjw@keri.re.kr (J.-W.P.)

**Keywords:** Li-ion batteries, binder-free electrodes, TiO_2_ nanotube arrays, electrochemical anodization, elemental doping

## Abstract

Vertically aligned Fe, S, and Fe-S doped anatase TiO_2_ nanotube arrays are prepared by an electrochemical anodization process using an organic electrolyte in which lactic acid is added as an additive. In the electrolyte, highly ordered TiO_2_ nanotube layers with greater thickness of 12 μm, inner diameter of approx. 90 nm and outer diameter of approx. 170 nm are successfully obtained. Doping of Fe, S, and Fe-S via simple wet impregnation method substituted Ti and O sites with Fe and S, which leads to enhance the rate performance at high discharge C-rates. Discharge capacities of TiO_2_ tubes increased from 0.13 mAh cm^−2^(bare) to 0.28 mAh cm^−2^ for Fe-S doped TiO_2_ at 0.5 C after 100 cycles with exceptional capacity retention of 85 % after 100 cycles. Owing to the enhancement of thermodynamic and kinetic properties by doping of Fe-S, Li-diffusion increased resulting in remarkable discharge capacities of 0.27 mAh cm^−2^ and 0.16 mAh cm^−2^ at 10 C, and 30 C, respectively.

## 1. Introduction

Titanium based oxides have drawn great attention in the lithium ion battery (LIB) world because of their superior thermal stability compared with the conventional graphite anode. Moreover, this class of active material shows other interesting features such as low cost, non-toxicity, and small volume change process (2–3%) during the lithium insertion and extraction, along with an excellent cycling life [1]. In general, bulk TiO_2_ shows a low theoretical capacity of 175–360 mAh g^−1^ and a low electrical conductivity. The electrochemical performance and the reversible capacity of titanium-based oxides mainly depend on their microscopic structure, morphology, and particle size [2]. Interestingly, the nanostructured titanium oxide leads to a superior capacity, longer cycling life, and higher rate capacity than bulk TiO_2_ [2,3].

TiO_2_ shows excellent safety and stability characteristics at the operation potential of 1.5 V vs. Li/Li^+^. Moreover, TiO_2_ has high electro-activity, strong oxidation capability, good chemical stability, high abundance, and structural diversity [4,5,6]. Where TiO_2_ based materials possess boosting the performance of battery, still they have limitations such as poor electrical conductivity and low Li-ion diffusivity, which result in poor electrochemical performance, thus hindering their practical application [4,5,6,7]. So far, many attempts have been made to compensate for this problem by means of using low-dimension (1 D, 2 D) TiO_2_ nanostructures composites [8,9]. 

The reduction of Ti^4+^→Ti^3+^ is accompanied by Li ion insertion/extraction into the oxide structure. Electrochemical fading in the crystal structure due to pulverizing of electrodes during volume expansion and reduction. In the advantage of taking forward TiO_2_ nanotubes (TNTs) as anodes, (i) the well-ordered electrode geometry reduces the ion diffusion path (ii) mechanical strain arising during Li ion insertion/extraction cycling can be accommodated, and (iii) therefore, the structural stability is maintained [10]. 

On the other hand, introducing the heteroatoms such as C, Nb, N, B, W, Sn, and Fe into TiO_2_ nanostructures is a promising way to stabilize these structures and improve the electron flow to accelerate the kinetics during electrochemical processes [11,12,13,14,15]. This will enhance the rate performance, cycling behavior, and specific capacity of TNTs in Li-ion battery application. However, the single substitutional doping in TiO_2_ mostly has a low thermodynamic solubility [16,17,18,19]. Therefore, a concept of co-doping idea is attempted in various studies as noted (including carbon, nitrogen; fluorine, nitrogen; chromium, nitrogen; and sulfur, nitrogen) in TiO_2_ nanoparticles as an anode material for Li-ion batteries [2,8,9,12,15,17,18,19]. In the previous studies, it has been demonstrated that the Fe-S co-dopant pairs can substantially narrow band gap and effectively modify the electronic structure of TiO_2_ [9,19]. However, so far only limited studies on doping of metal atoms in TNTs as anode for LIB are available [1,18,19,20].

In this study, vertically aligned self-organized TiO_2_ nanotubes are prepared by an electrochemical anodizing technique [21,22,23,24]. In conventional LIBs, up to 10% of “dead weights” loss occur when the additives such as polymeric binder and carbon conductor are used. However, these anodic TiO_2_ nanotube arrays directly formed on Ti can be used as an anode in LIB, so-called binder-free electrode. Furthermore, in order to enhance the electrochemical performance of TNTs, Fe-S co-doping was done by a wet immersion technique for boosting the electronic pathways and lithium ion diffusion coefficient, which closure with the result of greater storage performance.

## 2. Materials and Methods

### 2.1. Preparation of TNTs

**Figure 1:**
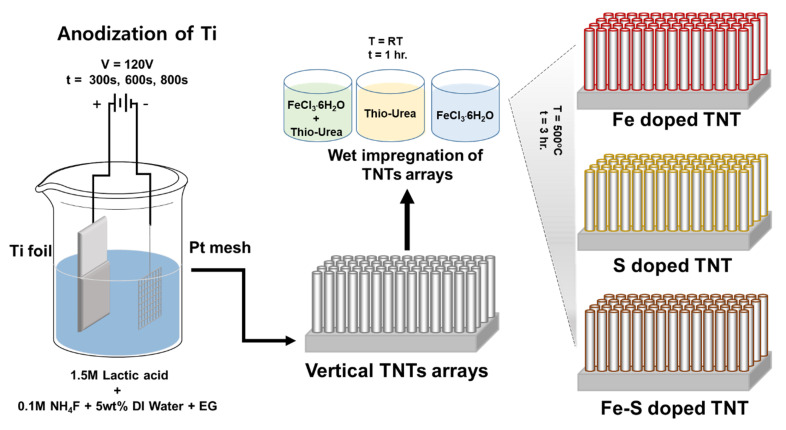
A schematic illustration of the preparation steps for Fe, S, and Fe-S doped TNTs.

Pre-cleaned Ti-foils (0.1 mm thick, 99.99% purity, Nilaco) were anodized in an electrolyte composed of 1.5 M lactic acid, 0.1 M ammonium fluoride and 5 wt. % deionized water in ethylene glycol [25]. The anodization was carried out in a two-electrode cell configuration: a Pt mesh was used as the counter electrode and the Ti foils were used as the working electrode. The anodization was conducted by using a high-voltage potentiostat (OPS-22101, ODA, Incheon, Korea) at a DC voltage of 120 V for 300 s, 600 s, and 800 s with the electrolyte temperature at 60 °C as shown in Figure 1. The obtained samples were rinsed in ethanol and dried in an oven at room temperature. 

### 2.2. Preparation of Fe-S co-doped TNTs

Fe, S, and Fe-S co-doped samples were prepared by using simple wet impregnation method as mentioned in a previous report [20]. For the solution preparation, 0.48 g of FeCl_3_·6H_2_O, 0.274 g of thiourea, mixture of 0.48 g of FeCl_3_·6H_2_O and 0.274 g of thiourea were dissolved in 20 mL of absolute ethanol under vigorous stirring until the mixture turned into clear. Subsequently, the prepared TNT samples were soaked in the solutions for 1 hr and then kept for drying at room temperature. In order to obtain the anatase crystalline TNTs, the dried samples were annealed at 500 °C for 3 h. using a tube furnace (XY-1400S, Hantech, Ulsan, Korea).

### 2.3. Materials Characterization

The morphological study of the synthesized materials was performed using a field-emission microscope (Hitachi FE-SEM S4800, Chiyoda City, Tokyo, Japan) equipped with energy dispersive spectroscopy as well. The structure and crystalline phase of the samples were characterized by X-ray powder diffraction (XRD, Philips, X-pert PRO MPD, Amsterdam, Netherlands) with Cu Kα (λ = 0.15406 nm). The electronic states of elements were characterized by X-ray photoelectron spectroscopy (XPS, K-Alpha + XPS System, Thermo Scientific, Loughborough, UK). XPS was conducted with a monochromatic Al Kα source (hʋ = 1486.6 eV) with a spot size of 400 μm.

### 2.4. Electrochemical Characterization

The vertically aligned TNTs arrays doped with Fe, S, and Fe-S grown on Ti foils was cut into disk (diameter of 14 mm) and used as the anode for the electrochemical tests. The weight of the Ti substrate and the active material (bare, Fe, S, and Fe-S doped TNT layers) are shown in Table S2. A coin half-cell with a polypropylene membrane separator (Celgard 2325, Celgard Inc., Charlotte, NC, USA) and a Li-metal foil (thickness = 500 μm, purity 99.9%) as the counter-electrode was used to evaluate the electrochemical performance. The fabricated TNT disks were directly used as a working electrode in the electrochemical cell without adding any conductive carbon or binder. The electrolyte was 1 M LiPF_6_ dissolved in 1:1:1, *v/v/v* mixture of ethylene carbonate, dimethyl carbonate, and ethyl methyl carbonate (EC: DMC: EMC) with 5% fluoroethylene carbonate (FEC). The amount of the used electrolyte was approximately 15 mL (g) and the ratios of electrolyte/active material are provided in Table S2. The assembled cells were galvanostatically cycled at different C-rates ranging from 0.2 C to 30 C in a potential range of 0.5–3 V using a multi-channel battery tester (MACCOR). Cyclic voltammetry (CV) was performed by using an electrochemical workstation (VMP3, Bio-Logic, Claix, France) with the same coin cell in the scan range of 0.5–3.0 V at a scan rate of either 1 mV s^−1^ or 0.5 mV s^−1^. Electrochemical impedance spectroscopy (EIS) test was performed by using an electrochemical workstation (VSP-300, Bio-Logic, Claix, France) in the frequency range of 10^−2^–10^+5^ Hz.

## 3. Results and Discussion

### 3.1. Morphology and Crystal Structure

**Figure 2:**
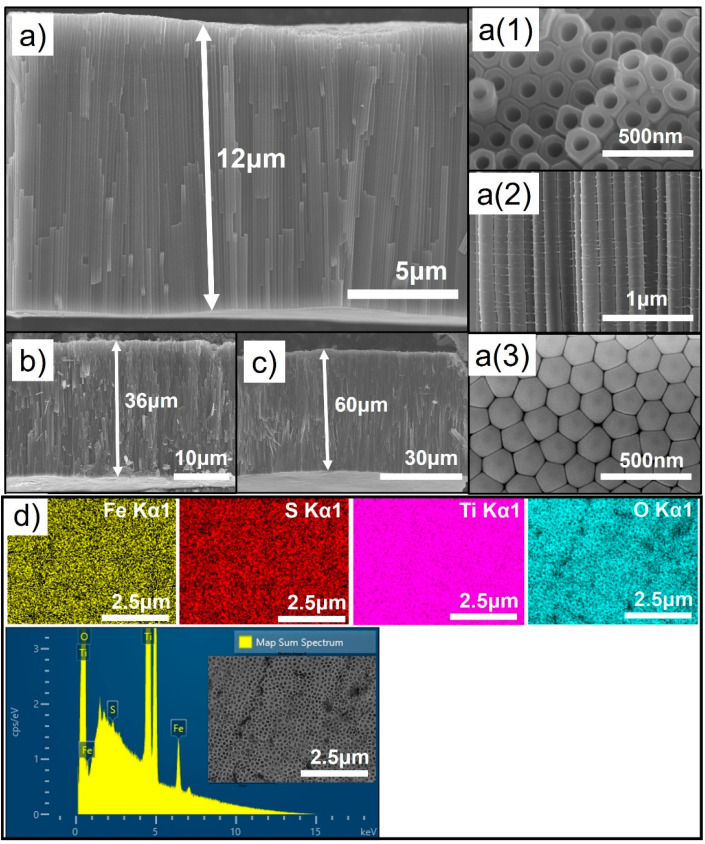
(**a**–**c**) shows FE-SEM images of as prepared TNTs. 12 μm, 35 μm, and 60 μm thick TNTs were prepared by anodization process at 120 V–60 °C for 300, 600, and 800 s, respectively. a(1), a(2), and a(3) shows the top, cross-section, and bottom view of 12 μm thick TNTs. (**d**) EDS results with elemental mapping of Fe-S doped TNTs. The inset SEM image shows the measured site.

Self-organized vertically aligned TiO_2_ nanotube layers were prepared via electrochemical anodization of Ti foils in the lactic acid added F^−^ ion containing electrolyte with 120 V at 60 °C for 300 s, 600 s, and 800 s to obtain layer thickness of 12 μm, 36 μm, and 60 μm, respectively. The length of the nanotubes mainly depends on the anodizing time, applied voltage, electrolyte temperature, etc [26]. Lactic acid added electrolyte was used to obtain a high layer thickness (i.e., a long length) in a relatively short time. This additive stabilized the TNTs formation and allowed the application of the high working voltage of 120 V and also the high temperature of 60 °C in the anodization process [25]. Figure 2a–c shows the FE-SEM results for the prepared TNTs with different anodizing time. The inset Figure 2a(1) shows the top view image which represents highly ordered TNT array having a uniform porosity, while Figure 2a(2) shows a cross-sectional view image which indicates the vertically aligned TNT array, and Figure 2a(3) shows the bottom view image of the hexagonally close packed TNT structure [27]. Figure 2d shows the SEM image of EDS mapping and elemental distribution result of Fe-S doped TNTs, where Fe, Ti, O peaks clearly shows the even distribution of elements.

**Figure 3:**
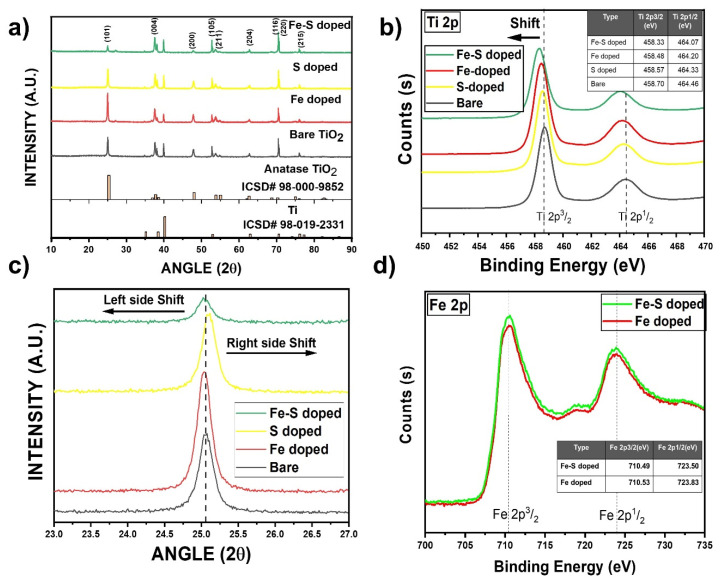
(**a**) XRD results for bare, Fe, S, and Fe-S doped TNTs. (**b**) Ti 2p XPS results for bare, Fe, S, and Fe-S doped TNTs showing a peak shift towards lower energies due to the presence of doped elements in the TNTs framework. (**c**) A zoomed in view of (101) XRD peak showing clear peaks shift for Fe, S, and Fe-S doped TNTs. (**d**) Fe 2p XPS results for Fe, Fe-S doped TNTs showing the incorporation of Fe in the TiO_2_ framework.

Figure 3a shows the XRD patterns for bare, S, Fe, and Fe-S doped TNTs. It reveals the existence of the anatase TiO_2_ for all the doped samples after annealing at 500 °C for 3 hrs with characteristic peaks at 24.025° (ICSD# 98-000-9852). A high peak intensity of the (004) orientation can be observed in all of the TNTs, which indicates a high percentage of (101) orientation in the growth direction of the TNTs (Figure S1a). Moreover, doping of different atoms did not affect the orientation except the intensity difference in (101) peak [25]. In Figure 3c, the diffraction peaks (101) of Fe and Fe-S doped TNTs shift to both lower angle and higher angle (Figure S6) are analyzed and the lattice parameters d_011_ are increased slightly from 0.450 nm for bare to 0.454 nm and 0.455 nm for Fe and Fe-S doped TNTs, respectively. This occurs due to the incorporation of Fe^3+^ (0.650 Å) having larger radius than Ti^4+^ (0.606 Å), suggesting that the Fe atoms have been successfully incorporated into the crystal structure of TiO_2_ [12,25]. However, in case of S doped TNTs, the peak shift is towards a higher angle. This phenomenon can be attributed towards the larger radius of Ti^4+^ (0.606 Å) than S^4+^ (0.370 Å) and S^6+^ (0.290 Å) [28]. However, the doping amount of S is kept very small in both Fe-S, S doped TNTs due to its high reactivity. Hence the effect is minimal in the lattice parameter change (Table S1). Similarly, the effect of the doping on crystallite size can also be observed. The crystallite size decreases significantly as a result of S, Fe-S doping, while in case of Fe doping the decrease in the crystallite size is not as drastic as the former ones. The average crystallite size, calculated from Scherrer equation of Fe-S, Fe, S doped, and bare TNTs are approximately 32.38 nm, 39.64 nm, 30.31 nm, and 45.93 nm, respectively (Table S1).

The electronic states of the dopants and the parent atoms in TNTs were analyzed by (XPS). As illustrated in Figure 3b, the most intense peaks at 458.70 eV and 464.46 eV correspond to Ti 2p3/2 and Ti 2p1/2 spin–orbit splitting peaks, respectively [29,30]. In the case of Fe, S, and Fe-S doped TNTs, the peaks shift towards lower energy. This confirms the presence of dopants which have replaced Ti and O atoms due to the difference in the ionization energy decreases [31,32]. In case of Fe-S doped TNTs, not only S^+2^ and S^+6^ replaced Ti but there is a small peak observed at 163.01 eV (Figure S1c) which shows that there is also S^−2^ replacing O^−2^ as well [33].

Figure 3d shows the XPS spectrum of Fe 2p, where the binding energies at 710.53 eV, 723.83 eV, and 710.49 eV, 723.50 eV corresponding to Fe 2p3/2 and Fe 2p1/2 can be easily observed in Fe doped and Fe-S doped samples. This indicates that the doped Fe is mainly in 3^+^ oxidation state. Due to the similarities of the radius between Fe^3+^ (0.650 Å) and Ti^4+^ (0.606 Å), Fe^3+^ can be incorporated into the lattice of TiO_2_ to form Ti–O–Fe bonds [12]. In the case of S, Fe-S doped TNTs, the doping amounts for S are extremely small as discussed previously and accordingly S2p spectra is not observed in case of S however, in case of Fe-S doped TNTs S peaks were observed. In Figure S1b, the O 1s XPS spectra of Fe-S doped TNTs are split as two peaks. The energy of the peak located at 529.56 eV is equal to the O 1s electron binding energy for TiO_2_. The other peak at 531.13 eV is ascribed to S–O–S bond, which confirms that the sulfur atoms replace a part of Ti sites [34] and in Figure S1c peak located at 163.01 eV [33]. This shows that sulfur is present in the form of S^2−^ by replacing O^2−^ which contradicts the previous report using thio-urea as a dopant precursor for sulfur [35]. This remarkable structural stability is expected to be conducive to reversible lithium storage with excellent cycle performances.

### 3.2. Electrochemical Performance 

**Figure 4:**
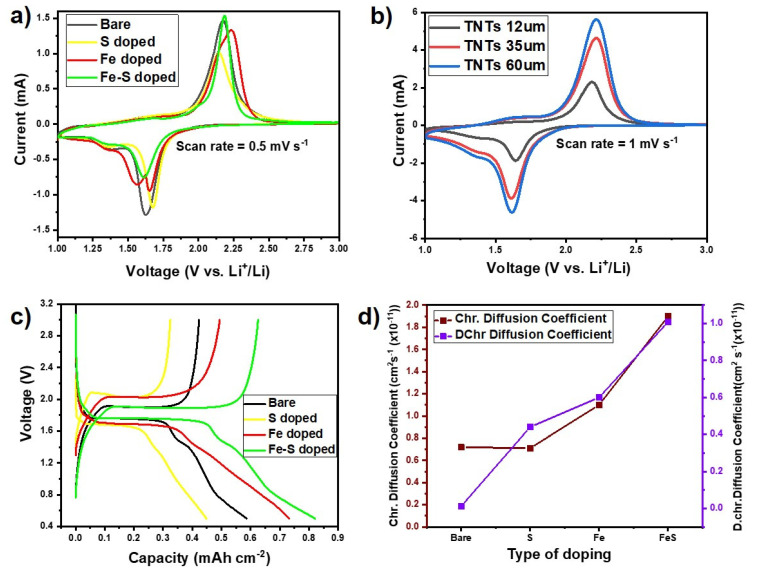
(**a**) Cyclic voltammograms of bare, Fe, S, and Fe-S doped TNTs with respect to Li metal at a scan rate of 0.5 mV s^−1^. (**b**) Cyclic voltammograms of TNTs with the different thickness of 12 μm, 36 μm, and 60 μm. (**c**) 1st charge-discharge profiles of bare, Fe, S, and Fe-S doped 12 μm thick TNTs at 0.5 C. (**d**) Diffusion coefficient values calculated from Randles–Sevcik equation for bare, Fe, S, and Fe-S doped TNTs.

In order to investigate the effect of different dopants in TNTs on the electrochemical performances, cyclic voltammetry experiments for bare, Fe, S, and Fe-S doped TNTs electrodes were conducted at 0.5 mV s^−1^ as shown in Figure 4a. Moreover, CV for TNTs with different thickness were also done 1 mV s^−1^ to investigate the effect of higher thickness on lithiation/delithiation of TNTs (Figure 4b). In principle, the reaction equation can be used to express the lithium insertion and extraction in anatase TNTs electrode:

TiO_2_ + nLi^+^ + ne^−^ ↔ Li_n_TiO_2_ (1)

All the sample electrodes (Figure 4a) exhibit peak couples at 2.14/1.63 V (bare TNTs), 2.17/1.67 V (S doped TNTs), 2.19/1.64 V (Fe doped TNTs), and 2.20/1.66 V (Fe-S doped TNTs) corresponding to the transition of Li poor α-Li_x_TiO_2_ (0.01 < x ≤ 0.21) with anatase structure to the orthorhombic β Li_x_TiO_2_(x~0.55) phase, their positions are in good agreement with those reported in the literature [36]. In case Fe-doped TiO_2_ in reduction peaks, there is an additional reaction happens due to the over potential of phase transformation from TiO_2_ to Li_x_TiO_2_. In order to reduce the polarization effect happens between the electrode and electrolyte interface, the Fe-doping in TiO_2_ will reduce this effect and enhance the electronic conductivity, which results that further enhancement in electrochemical storage [37]. Moreover, there is a small peak pair visible at 1.62 V (lithiation) and 1.4 V (delithiation), which corresponds to a second phase change to fully lithiated LiTiO_2_ [36]. This feature is more prominent in the case of higher-length tubes as shown in Figure 4b. Moreover, it can be seen that upon delithiation peak broadening is observed which is a characteristic for self-oriented TNTs. In contrast to bare, Fe, S, and Fe-S TNTs show a prominent second phase transition upon delithiation. 

The diffusion coefficient of lithium ion during the Li^+^ intercalation/de-intercalation processes can be calculated according to Randles–Sevcik equation [38]. Figure 4d and Figure S4b shows the calculated diffusion coefficient values for doped TNTs and elongated TNTs. The Li^+^ diffusion increases by doping of Fe, S, Fe-S. In case of doping Li^+^ ion diffusion increased from 0.75 × 10^−11^ cm^2^ s^−1^(bare TNTs) to 0.13 × 10^−10^ cm^2^ s^−1^ (Fe-S doped TNTs) for de-intercalation and 0.2 × 10^−11^ cm^2^ s^−1^ (bare TNTs) to 0.12 × 10^−10^ cm^2^ s^−1^ (Fe-S doped TNTs) for intercalation. It should be noted that Li^+^ diffusion is greatly improved by co-doping with both Fe and S in anodic TNT’s framework. 

Moreover, preferentially oriented tubes are derived by anodization process and even directly use of these TNTs in LIB increases the charge/discharge capacities [39]. However, these performances are considerably enhanced by co-doping with Fe-S in the TNT structures. In addition, the CV curves at a scan rate of 0.5 mV s^−1^ as shown in Figure S2 are stable with almost overlaps from the second cycle, which indicate well posited dopant in the structures and excellent stability for Fe, S, and Fe-S doped TNT electrodes.

Galvanostatic charging/discharging was carried out at C-rate of 0.5 C in the range of 0.5–3.0 V vs. Li^+^/Li^−^, Initial discharge capacities for bare, S, Fe, and Fe-S doped TNTs anodes came out to be around 0.6 mAh cm^−2^, 0.45 mAh cm^−2^, 0.75 mAh cm^−2^, and 0.84 mAh cm^−2^ (Figure 4c). It can be seen that the discharge capacities for S doped TNTs are lower when compared to others. This is because of the reaction of sulfur with Li to form Li_x_S which in result decreases the discharge capacity [40].

However, further in this study it will be shown that this does not affect the cycling behavior of TNTs but increase cyclability of S doped TNTs. It can also be observed that the charge (1.9 V) and discharge (1.65 V) plateaus for Fe-S doped TNTs show lowest and highest among the other electrodes, respectively. This indicates the lowest electrochemical polarization and the best charge/discharge energy density of Fe, S doped TNTs anode. Moreover, the specific capacity is proportional to time interval (i.e., scan rate) of charge/discharge [11,40]. The sloping region for the Fe-S doped TNTs electrode below the plateau corresponds to the pseudo-capacitive lithium storage in the surface area [41].

C = i/(dV/dT)(2)

Hence the sloping region will indicate the pseudo-capacitive lithium storage in the structures of TNTs. This shows that the shallowest slope (dV/dc) observed with the Fe-S doped TNTs electrode represents the highest capacitance values as compared to the steep slopes from the other three kinds of electrodes. The calculated capacitance values of bare, Fe, S, Fe-S doped TNTs are 46.5%, 52%, 50%, and 54%. The corresponding values shows the Fe-S doped TNTs have the high pseudo-capacitance behavior [42], which leads to increase in the capacity of TNTs from 0.6 mAh cm^−2^ for bare TNTs to 0.81 mAh cm^−2^ for Fe-S doped TNTs. It is noted that the capacity values are reported with areal unit (mAh cm^−2^) due to the reason that the mass loading in anodized TiO_2_ nanotubes (as an active materials) directly grown on Ti substrates (as a current collector) cannot be precisely measurable with gravimetric unit measurement since the electrode itself together with the TiO_2_ active materials and the Ti current collector is one solid body and, moreover, with the areal capacity we can further discuss with our previous study of anodic TiO_2_ in LIBs [43,44,45,46,47]. However, in Figure S3, we provide both areal and “estimated” gravimetric capacities for bare and Fe-S doped TNTs. It is clear that Fe-S doped TNTs show a prominent cycling as compared to bare TNTs. In Figure S5, we show the high rate of gravimetric capacity for different C-rates.

**Figure 5:**
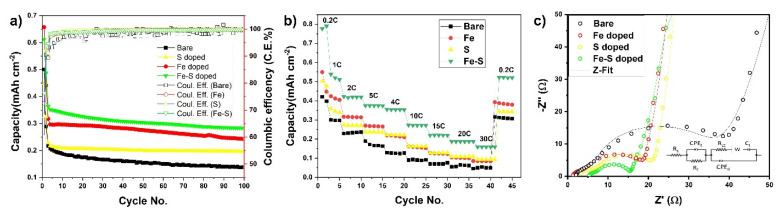
(**a**) Cycle vs. discharge capacity curves for bare, Fe, S, and Fe-S doped TNTs cycled at 0.5 C. (**b**) Rate capablity of bare, Fe, S, and Fe-S doped TNTs. (**c**) EIS curves of bare, Fe, S, and Fe-S doped TNTs after 100 cycles.

Moreover, in case of Fe, S, and Fe-S after 60 cycles (Figure 5a), they show higher discharge capacities of 0.24 mAh cm^−2^, 0.19 mAh cm^−2^, and 0.28 mAh cm^−2^ as compared to 0.13 mAh cm^−2^ of bare TNTs. Fe-S doped and bare TNTs were further cycled to 100. In the cycling results, it was found that Fe-S retained 85% discharge capacity (taken after 3rd cycle) as compared to bare TNTs with 65% (i.e., doping of Fe, S together increases the capacity retention and increased capacitive properties). Moreover, columbic efficiency in the 1st cycle is 76% for Fe-S doped TNTs and 60% for bare TNTs. These high irreversible capacities can be because of the higher length of the tubes and side reactions with sulfur and absorbed moisture due to high specific surface area [48]. However, this is redeemed in the 2nd cycle with 90.9% for Fe-S and 93.8% for bare TNTs. The decrease in columbic efficiency for Fe-S as compared to bare TNTs can be because of reaction of Li with S to form Li_x_S, however, this product is redox active [40]. Hence, this will help in further cycling and increase the electrochemical stability with the high capacity retention as shown in Figure 5a.

Figure 5b shows rate capability cycling results for bare, Fe, S, and Fe-S doped TNTs. The cells were charged at 0.2 C and discharged at different C-rate. It is evident that Fe-S doped TNTs shows the best rate capabilities at various C-rates compared to charge-discharge study is applied from 0.2 C to 30 C. All the doped TNTs showed exceptional rate capabilities at higher rates as compared to bare TNTs. Fe-S doped TNTs show 0.27 mAh cm^−2^ at 10 C and 0.14 mAh cm^−2^ at 30 C for two different C-rates as compared to 0.07 mAh cm^−2^ at 10 C and 0.03 mAh cm^−2^ at 30 C of bare TNTs. It is important to note that Fe-S doped TNTs retained the reversible specific capacities of 0.51 mAh cm^−2^ at 0.2 C as well when cycled again. These results clearly validate that the Fe-S doped TNTs electrodes exhibit superior lithium storage properties with prolonged cycle life and great rate capability for the fast discharge process.

To further understand the origin of the superior electrochemical properties of doped TNTs and to study differentiate kinetic and thermodynamic properties of the TNTs electrodes upon lithiation and delithiation, electrochemical impedance spectroscopy (EIS) was employed in the frequency range of 10^−2^–10^+5^ Hz. This allows for differentiation of processes taking place at different time scales during Li insertion and extraction. Figure 5c shows us the EIS spectrum and the fitting circuit of bare, Fe, S, and Fe-S doped TNTs after 100 cycles. In all of the observed EIS spectrums, at the highest frequencies, a depressed semicircle can be observed which corresponds to the parallel combination of surface film resistance R_f_ and surface film capacitance C_f_ [48]. These thin surface films originate from the decomposition of compounds from the carbonate-based (EC, DMC) LiPF_6_ electrolytes [49]. The second medium-frequency semi-circle relates to the interfacial charge transfer, represented by the charge transfer resistance, R_ct_, and the double-layer capacitance, C_dl_, in parallel insertion [7]. It can be seen that, after 100 cycles R_ct_ values decreased when compared to bare TNT electrodes. R_ct_ values of 20.78 ohm, 18.49 ohm, and 15.54 ohm were achieved for Fe, S, and Fe-S doped TNTs as compared to 38.47 ohm for bare TNTs. However, in the nanotube system, the charge transfer may not be the only rate-determining step; therefore, it is also necessary to consider solid state diffusion in the bulk material [50]. This shows that both the increase in Li^+^ diffusion and charge transfer resistances of doped TNTs increased the cycling and rate cycling properties. In order to observe the difference between before and after cycling effect in Fe-S doped TNTs, EIS was done before cycling of Fe-S doped TNTs samples as well. Figure S4a shows the EIS spectrum and fitting of before and after 100 cycles. It can be observed that R_ct_ values for Fe-S doped TNTs decreased from 269 ohm to 15.54 ohm after 100 cycles. During lithiation, in case of TiO_2_, fully lithiated Li_1_TiO_2_ forms with higher charge resistance, moreover, the decomposition of LiPF_6_ electrolyte to LiF and Li_2_CO_3_ also increase the charge transfer resistance between electrode/electrolyte interfaces. However, by the introduction of Fe and S as co dopants the fully lithiated phase transition is improved by decreasing the charge transfer resistance and as a result higher reversibility during lithiation and de-lithiation is achieved [51]. This incredible increase in electronic conductivity and charge transfer can be attributed towards the modified electronic structure resulting in exceptional properties and uniform pathways for Li^+^ intercalation/de-intercalation. Anodic TNTs improved the structural as well as the directional properties compared to wet synthesized TNTs. TiO_2_ Nanoparticle of 0D material, which are smaller in size shortens the electronic pathways and increased the Li^+^ diffusion in the lattice. Similarly, the 2D nanotube shows relative high surface area which provides more active surface sites compared to TiO_2_ nanocrystals. It further enhances the fast lithium ion transfer between electrode and electrolyte, which leads to closure in deducing the charge transfer as shown in EIS results. Moreover, the increase in tubular size to 12 μm as compared to conventional small sized tubes helped in increasing the discharge capacities [20]. Doping of Fe-S, Fe, and S in TNTs increased the interlayer spacing, thus favoring Li^+^ intercalation/de-intercalation as well. Fe-S doped TNTs showed best results with exceptional increase in cycling and rate cycling discharge capacities at exceptionally higher rates.

## 4. Conclusions

As a binder free anode, vertically aligned TNT layers with different dopants are prepared and performed as LIBs anodes. Co-doping of Fe and S proved to be fruitful, and amplified the storage performance to a comparable level to bare TNTs with notable high rates and cycling stability. This was attributed to boosting the Li^+^ diffusion (D) and deducing the charge transfer resistance (R_ct_). Moreover, the enhanced performance is due to the well-ordered geometry with a high aspect ratio and improved crystallinity of the TNT anodes.

## Supplementary Materials

The following are available online at https://www.mdpi.com/article/10.3390/nano11112924/s1, Figure S1: (a) XRD results of amorphous and crystalline TNTs. The diffraction peak of TiO_2_ amorphous annotate Ti metal. (b) XPS spectra for O 1s of Fe-S doped TNTs. (c) XPS spectra for S+4and S-2 of Fe-S doped TNTs, Figure S2: (a–d) Cyclic voltammetry curves at a scan rate 0.5mVs-1 for Fe-S, Fe, S doped TNTs and bare 12μm TNTs, respectively, Figure S3: Gravimetric and areal capacity retention with cycling of Fe-S doped and bare TNT anodes discharged at C-rate of 0.5 C, Figure S4: (a) EIS spectrum of before and after cycling for Fe-S doped TNTs. (b) Diffusion coefficient values as calculated using Randles–Sevcik equation for doped and elongated TNTs, respectively, Figure S5: High rate gravimetric capacities for bare, Fe, S, and Fe-S doped TNT’s discharged at different C-rates, Figure S6: A zoomed in view of (105) and (211) XRD peak showing clear peaks shift for Fe, S, and Fe-S doped TNTs, Table S1: Calculated lattice parameters and crystallite sizes of TNTs, Table S2: Weight of Ti substrate and active material of TiO_2_ nanotube layers and ratio of electrolyte/active material
