# Supplementary material for: Vertically Aligned Binder-Free TiO2 Nanotube Arrays Doped with Fe, S and Fe-S for Li-ion Batteries
Source: Nanomaterials (Basel). 2021 Oct 31;11(11):2924. doi: 10.3390/nano11112924 (PMC8623386; doi:10.3390/nano11112924)
Supplement: Supplementary file 1 [file nanomaterials-11-02924-s001.zip › nanomaterials-1405125-supplementary.pdf]

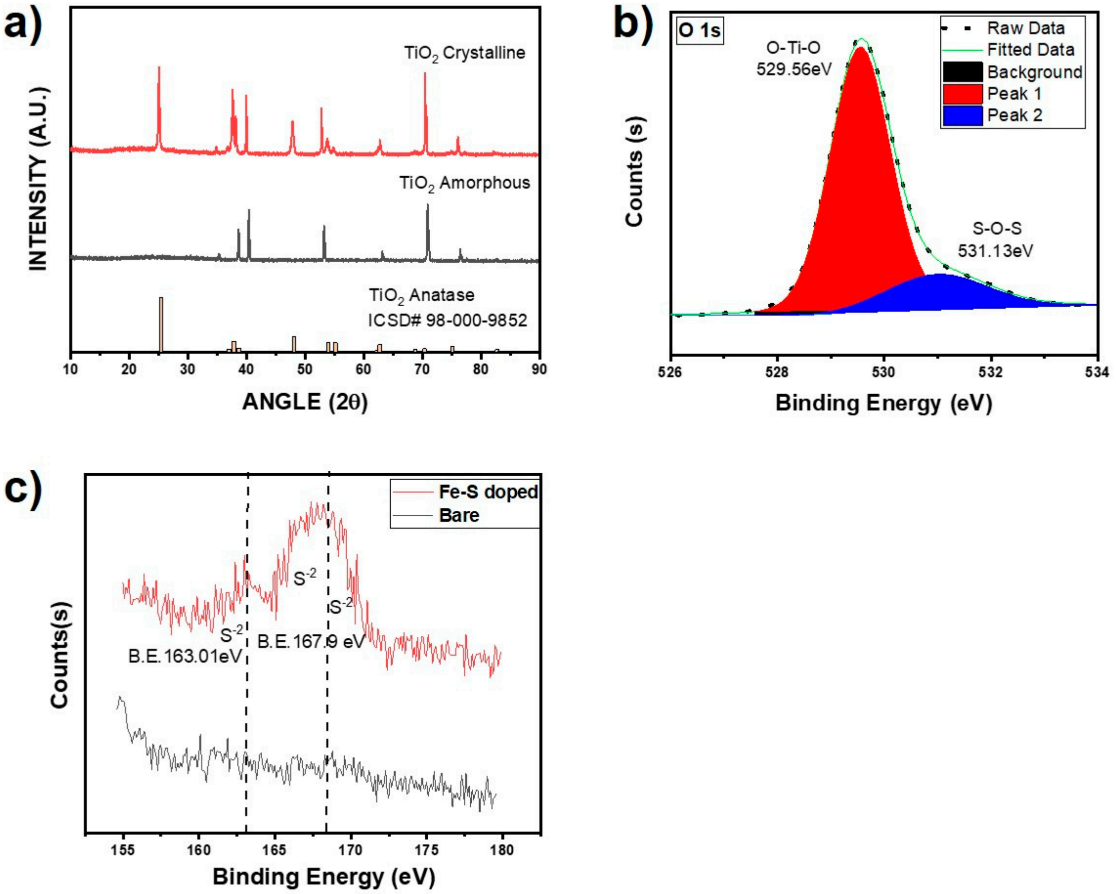

Figure S1. (a) XRD results of amorphous and crystalline TNTs. The diffraction peak of TiO2 amorphous annotate Ti metal. (b) XPS spectra for O 1s of Fe-S doped TNTs. (c) XPS spectra for $S^{+4}$and S^{-2} of Fe-S doped TNTs.

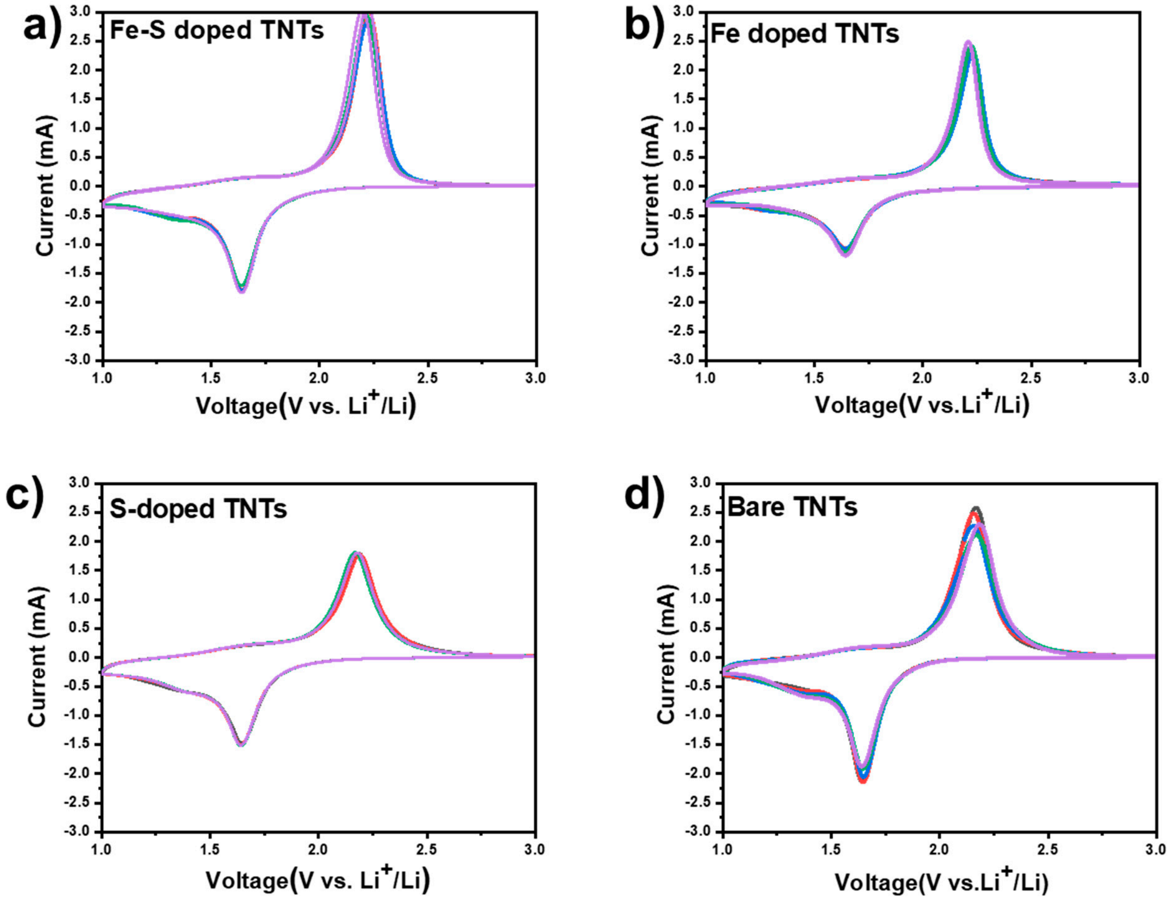

Figure S2. (a), (b), (c) and (d) Cyclic voltammetry curves at a scan rate $0.5 mVs^{-1}$ for Fe-S, Fe, S doped TNTs and bare 12μm TNTs, respectively.

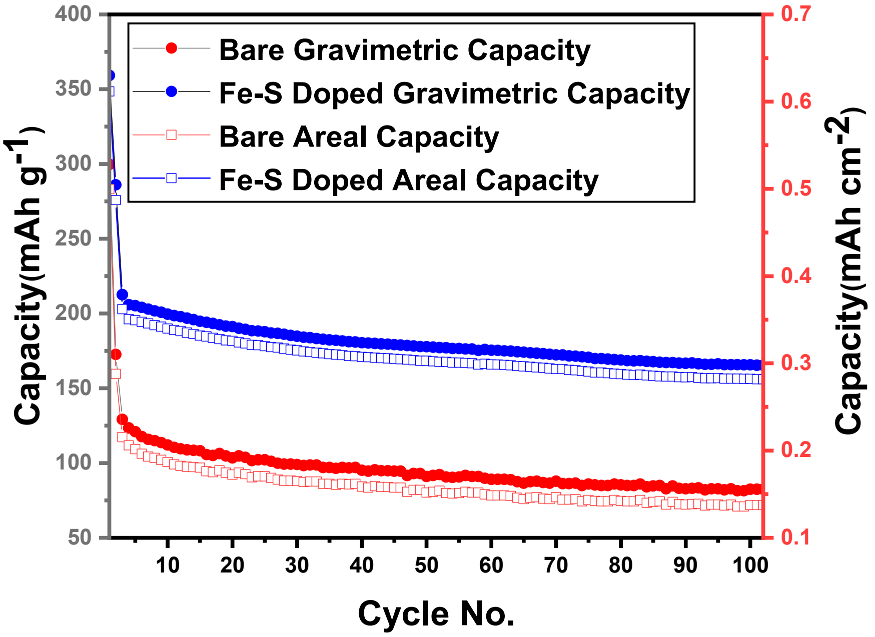

Figure S3. Gravimetric and areal capacity retention with cycling of Fe-S doped and bare TNT anodes discharged at C-rate of 0.5 C.

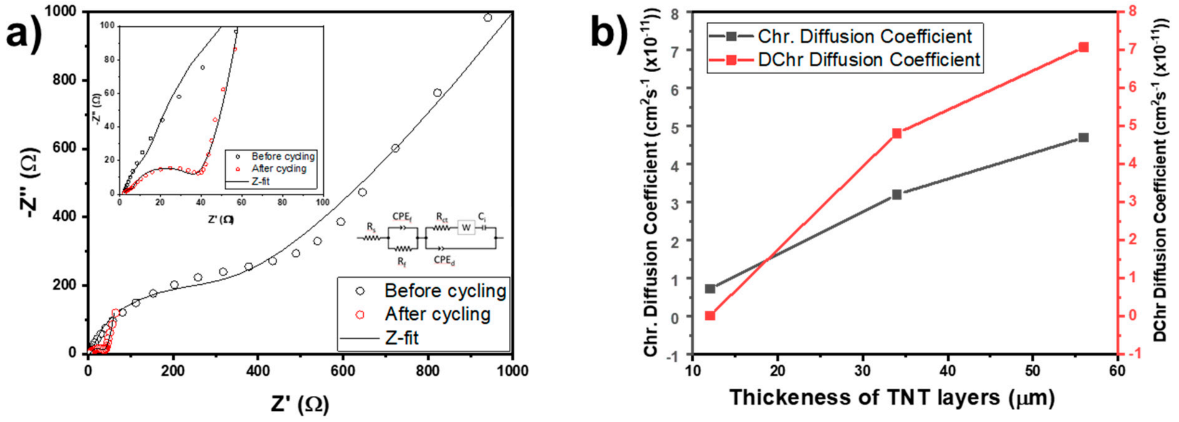

Figure S4. (a) EIS spectrum of before and after cycling for Fe-S doped TNTs. **(b)** Diffusion coefficient values as calculated using Randles–Sevcik equation for doped and elongated TNTs, respectively.

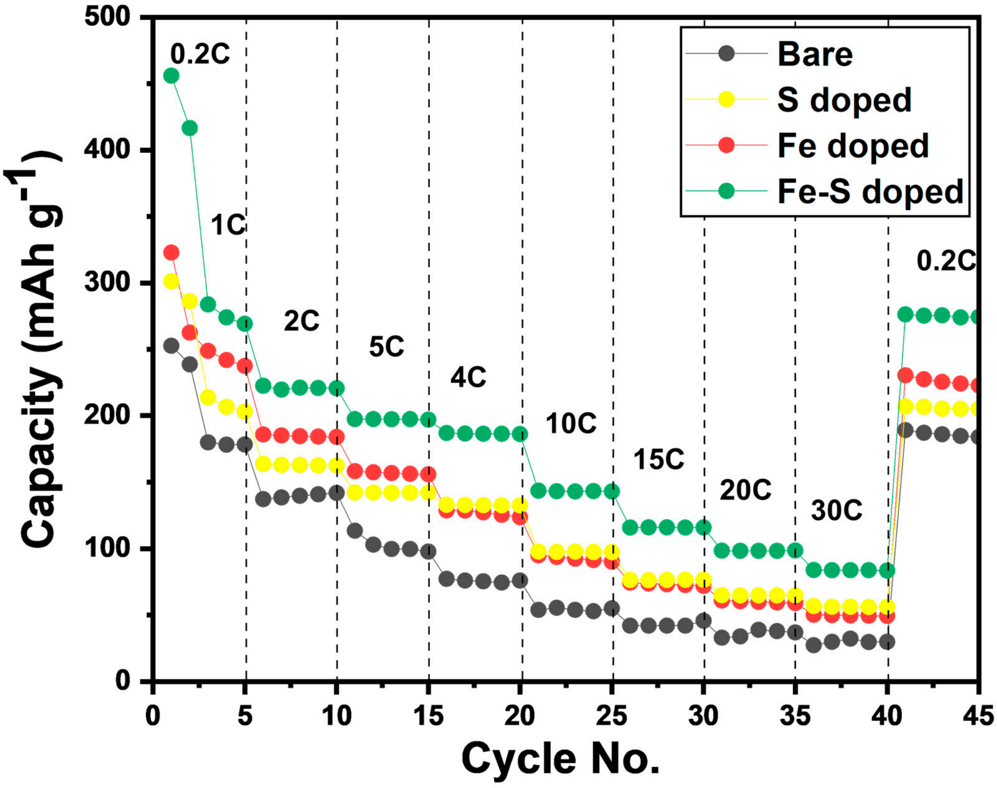

Figure S5. High rate gravimetric capacities for bare, Fe, S, and Fe-S doped TNT's discharged at different C-rates.

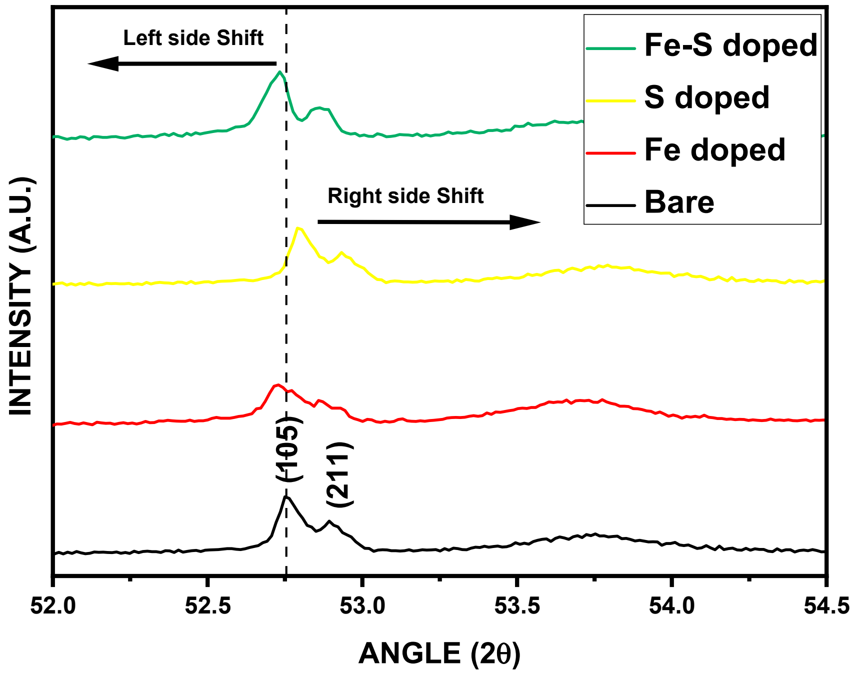

Figure S6. A zoomed in view of (105) and (211) XRD peak showing clear peaks shift for Fe, S, and Fe-S doped TNTs.

Table S1. Calculated lattice parameters and crystallite sizes of TNTs.

Material	Lattice constant (d_{011})[a]	Avg. crystallite size[b]
Bare	0.350 nm	45.93 nm
Fe doped	0.454 nm	39.63 nm
S doped	0.452 nm	30.31 nm
Fe-S doped	0.455 nm	32.38 nm

[a] calculated via Bragg's equation. [b] calculated via Scherrer equation

Table S2. Weight of Ti substrate and active material of TiO_2 nanotube layers and ratio of electrolyte/active material.

Material	Ti substrate (g)	Active material (g)	Electrolyte / Active material ratio
Bare	0.05540	0.01309	13.84
Fe doped	0.05623	0.01337	13.55
S doped	0.05499	0.01351	13.41
Fe-S doped	0.05711	0.01337	13.55

· Ti-foils (0.1 mm thick, 99.99 % purity, Nilaco)

· The diameter of electrode (substrate + active material) = 14mm

· The amount of battery electrolyte = 0.1812 g (0.15ml)
